# Influence of Shortened Tongue Frenulum on Tongue Mobility, Speech and Occlusion

**DOI:** 10.3390/jcm12237415

**Published:** 2023-11-29

**Authors:** Aldona Dydyk, Marta Milona, Joanna Janiszewska-Olszowska, Marzena Wyganowska, Katarzyna Grocholewicz

**Affiliations:** 1 Specialized Dental and Medical Offices MEDICO, 70-781 Szczecin, Poland; 2 Department of Hygiene and Epidemiology, Pomeranian Medical University, 70-204 Szczecin, Poland; 3 Department of Interdisciplinary Dentistry, Pomeranian Medical University, 70-204 Szczecin, Poland; 4 Department of Dental Surgery, Periodontology and Oral Mucosa Diseases, Poznan University of Medical Sciences, 60-806 Poznan, Poland

**Keywords:** ankyloglossia, occlusion, swallowing, tongue frenulum, tongue mobility, speech

## Abstract

(1) Background: The incidence of ankyloglossia is 0.02–10.7%. Its effect on selected dysfunctions has been described; however, no studies report its impact on several disorders in a group of subjects. The aim of this study was to assess the effect of ankyloglossia on swallowing, speech, occlusion and periodontium. (2) Methods: The study group consisted of 86 patients with ankyloglossia, and the control group (n = 86) had a normal tongue frenulum. Type of swallowing, tongue mobility, speech, occlusion and periodontium were assessed. (3) Results: Ankyloglossia pertained to 75.6% patients with infantile swallowing and 41.3% patients with mature swallowing. Limited tongue mobility was found in 29.4% subjects with moderate ankyloglossia and 70.6% subjects with severe ankyloglossia. All subjects with mild ankyloglossia and all the controls had normal tongue mobility. The relationship between dysglossia and ankyloglossia severity was statistically significant. Malocclusion or crowding was diagnosed in 62% subjects with ankyloglossia and 21.6% subjects in the control group. No periodontal abnormalities were found in any subject. (4) Conclusions: (1) A short tongue frenulum negatively influences swallowing and is associated with an “infantile swallowing pattern”. (2) Moderate or severe ankyloglossia significantly limits tongue mobility. (3) A short tongue frenulum negatively influences speech. (4) Ankyloglossia is associated with higher prevalence of malocclusion.

## 1. Introduction

The concept of ankyloglossia was first used in 1963 by Wallace and defined as a condition where the tip of the tongue cannot be extended further than the incisal edge of the lower incisors due to a short frenulum [1]. Since then, many diagnostic criteria have been developed in the literature, as well as many new terms for frenulum anomalies, such as tongue-tie, hypertrophic frenulum, thick frenulum, muscular frenulum, fibrotic frenulum, frenulum with anterior insertion, short frenulum and short frenulum with anterior insertion [2,3]. The incidence of ankyloglossia ranges from 0.02 to 10.7% and is 1.5–3 times more prevalent in boys than in girls [4,5,6,7]. It is significantly higher in neonatal studies (1.72–10.7%) than in older children (0.02–2.08%) [8]. It is likely that mild forms of shortened frenulum self-correct with tongue growth between 6 months and 6 years of age, explaining this discordance. Ankyloglossia most often occurs as an isolated defect, although rare, and it can also coexist with other congenital craniofacial defects, such as cleft lip, and syndromes, including Smith–Lemli–Opitz, Beckwith–Weidmann, Simpson–Golabi–Behmel, Kindler, van der Woude syndrome and X-linked cleft palate, or oropalatal dysplasia Bettex–Graf [9,10,11,12,13,14,15,16,17]. Family history confirming the presence of a short frenulum in these cases is recorded in 21–41% of cases [4,18]. The inheritance of isolated ankyloglossia may be X-linked [8,18].

Ankyloglossia develops when the tongue frenulum does not retract during the lengthening of tongue muscles in fetal life [19]. Normally, frenulum reduction begins in the 5th week of fetal life and is based on apoptosis, i.e., selective degeneration of the cells that make up the frenulum. It causes a “release” of the tongue from the floor of the mouth. Sometimes this process is disturbed, and the frenulum does not retract but remains thick and short, and sometimes, it completely connects the body of the tongue to the floor of the mouth, immobilizing the tongue.

Ruffoli et al. [20] used the measurement of the frenulum length itself and the assessment of the maximum mouth opening with the tongue placed on the incisive papilla, measured as the distance between the incisal edge of the lower incisor and the incisal edge of the corresponding upper incisor. The authors of the cited study developed a method for diagnosing ankyloglossia by listing morphofunctional symptoms such as limited tongue mobility, speech problems, oral cavity floor tension, swallowing pattern, changes in occlusion and tongue resting position. They showed that the direct measurement of the frenulum length, i.e., from the floor of the mouth to the point of penetration of the frenulum into the lower surface of the tongue, and the indirect measurement, i.e., between the incisal edges of the upper and lower incisors, are equivalent. The authors diagnosed ankyloglossia when the frenulum length was less than 20 mm or the interincisal distance was less than 23 mm and defined three levels of severity of ankyloglossia.

The literature describes the effect of ankyloglossia on selected dysfunctions of the stomatognathic system, but no studies could be found reporting on the influence of ankyloglossia on the occurrence of several disorders in the same group of subjects. Often, the studies published lack control groups without ankyloglossia.

The aim of the present study was the assessment of the impact of a short lingual frenulum on tongue function during swallowing, mobility, speech, malocclusion and periodontal status.

## 2. Materials and Methods

The study was approved by the Bioethics Committee at Pomeranian Medical University in Szczecin, decision reference No. 10/KB/V/2013.

The subjects were 172 patients (96 females and 76 males), at the ages between 5 and 60 (average of 33.3 years), of which 86 with ankyloglossia (study group) and 86 with normal tongue frenulum length (control group). Table 1 presents the distribution of the study and control groups according to sex.

The inclusion criteria for the study comprised the following:

- No history of frenulectomy (surgical excision of the frenulum), frenotomy (the snipping and slightly relocation of the frenulum), frenulotomy (transverse incision of the frenulum at its base) or any soft tissue surgery in the oral cavity (except tooth extractions or chiseling);
- No history of speech or swallowing therapy;
- No previous or ongoing orthodontic treatment;
- Preserved supporting zones in the lateral sections (with possible single teeth missing);
- Present permanent lower central and lateral incisors;
- Lack of calculus on the lingual surfaces of the lower anterior teeth.

Patients or their guardians were informed about the study and gave their informed consent to participate. In all subjects, the length of the tongue frenulum was measured three times, using a geometric compass, from the site of contact between the frenulum fold and the oral mucosa to the site where the fold of the mucosa of the tongue frenulum penetrates into the lower surface of the tongue body. The examination was carried out in a sitting position with the head positioned parallel to the Frankfurt plane. The length of the frenulum was taken as the arithmetic mean calculated from the three measurements.

**Figure 1:**
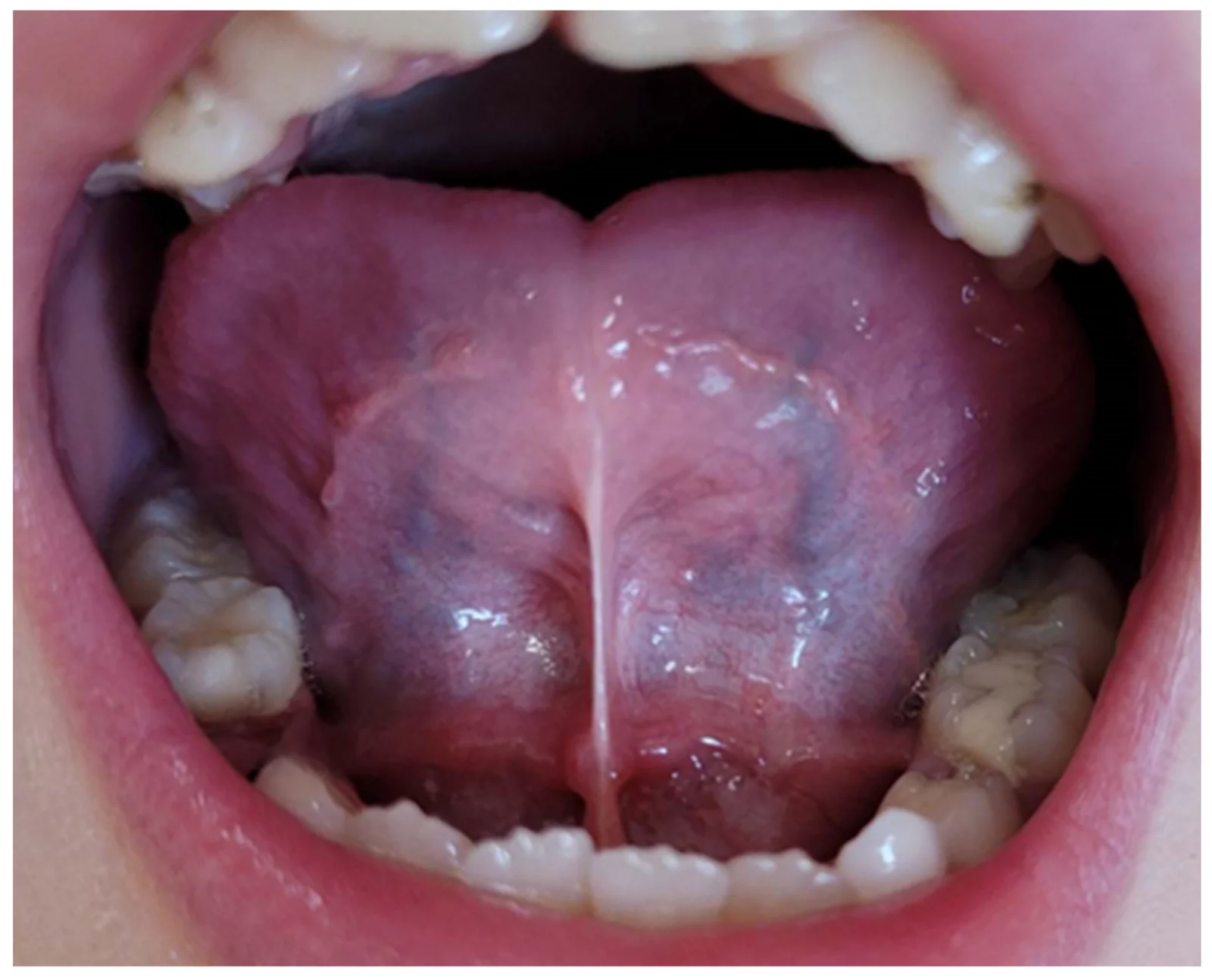
Mild ankyloglossia; own photography.

**Figure 2:**
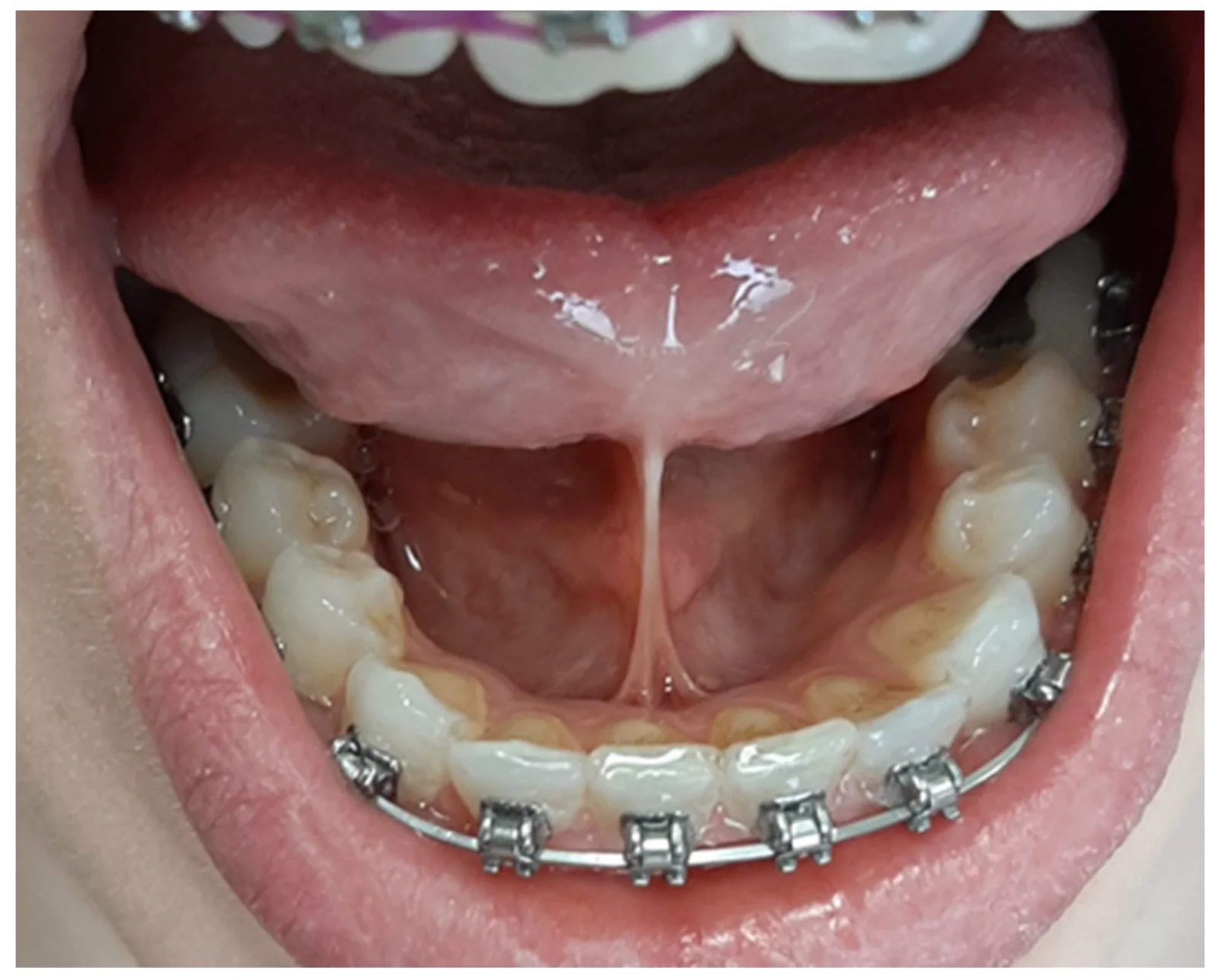
Moderate ankyloglossia; own photography.

**Figure 3:**
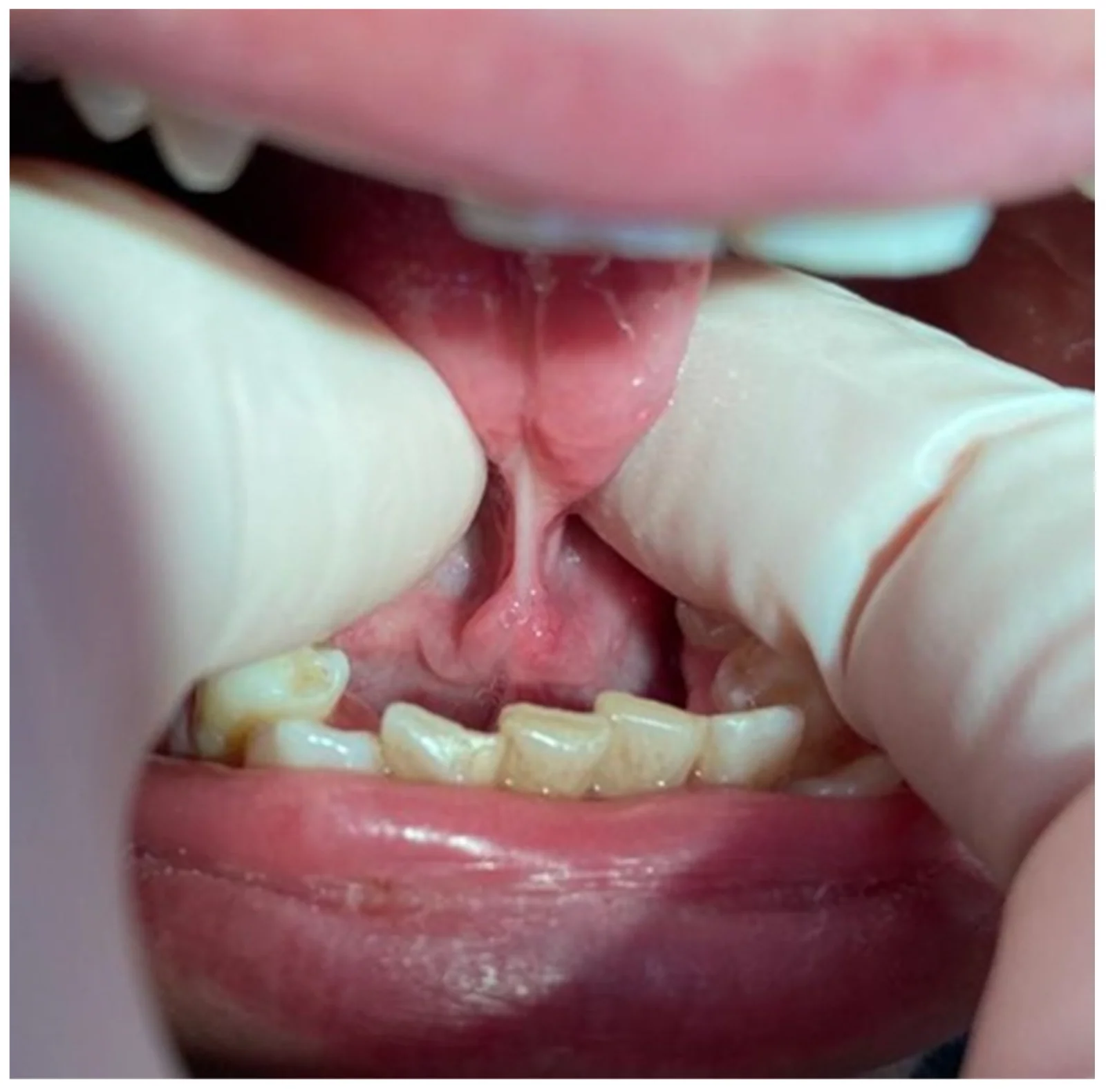
Severe ankyloglossia; own photography.

Depending on the length of the frenulum, the severity of the disorder was determined according to the method described by Ruffoli et al. [20] as follows:

- Frenulum length of 19–16 mm: mild ankyloglossia; an example is shown in Figure 1.
- Frenulum length of 15–8 mm: moderate ankyloglossia; an example is shown in Figure 2.
- Frenulum length of less than 7 mm: severe ankyloglossia; an example is shown in Figure 3.

A frenulum length of 20 mm or more was considered normative.

The type of swallowing was assessed during swallowing, with upper and lower lips slightly parted by the tips of the examining doctor’s index finger and thumb, which made it possible to assess the position of the tip of the tongue during the oral phase of swallowing. An infantile swallowing pattern was stated when the interposition of the tongue between the teeth during swallowing was found; a mature pattern, when the retroincisive position of the tongue in the region of the papilla was observed; a mixtus pattern, when infantile and mature swallowing tended to overlap.

The functional assessment of tongue mobility was carried out using functional tests, which were carried out identically in all the subjects, and these were the following movements:

1. Lifting the tongue up towards the palate;
2. Lowering the tongue;
3. Touching the incisive papilla with the tip of the tongue;
4. Touching the inside of the cheeks on both sides with the tip of the tongue;
5. Moving the tongue sideways to the corners of the mouth;
6. Protrusion of the tongue on the chin.

If a subject was able to perform all these movements, the mobility of the tongue was considered normal; if at least one of the above-mentioned movements could not be performed, tongue mobility was considered limited.

The presence or absence of dysglossia in the range of the Polish “r” /r/ sound (rhotacism) was determined by evaluating the pronunciation of three Polish words: rybak /ˈrɨ.bak/, strumień /ˈstru.mjɛɲ/, trąbka /ˈtrɔmp.ka/ (meaning fisherman, stream and trumpet, respectively, in English). The presence or absence of dysglossia in the range of the Polish sounds “s” /s/ and “sz” /ʂ/ (lisping) was determined based on the assessment of the pronunciation of the following three Polish words: sześć /ʂɛɕt͡ɕ/, szesnaście /ʂɛɕˈnaɕt͡ɕɛ/, koszyk /ˈkɔʂɨk/ (meaning six, sixteen and basket, respectively, in English).

To evaluate the occlusion, the subject was asked to clench the teeth in the position of maximum intercuspation. The presence or absence malocclusions, such as distal occlusion, mesial occlusion, open bite and crossbite, as well as dental crowding, was assessed.

The periodontal examination was aimed at determining the presence of normal connective tissue attachment within the marginal gingiva around the lingual surfaces of the crowns of the lower central incisors or the presence of recession or dehiscence of the marginal gingiva in this area.

The results were recorded in a unified patient examination chart developed for the purpose of the study.

As the variables analyzed were non-parametric, no sample size analysis could be performed for the present study. However, the authors are strongly convinced that a group of 172 patients is sufficient to find significant correlations and draw clinically relevant conclusions.

Statistical analysis was performed in the statistical language R [21], using the packages readxl [22], sjmisc [23], sjPlot [24], report [25] and rcompanion [26]. The level of significance was set to α = 0.05 for all statistical tests. Variables on the nominal, ordinal scale were analyzed in pairs in the form of contingency tables with frequency indication. The relationship between the variables was tested using Fisher’s exact test, additionally, measures of the strength of Cramer’s V or phi Yule φc relationship were calculated. For this purpose, the tab_xtab() method of the {sjPlot} package was used. In the case of the significance of the test with more than two groups, the significance between individual pairs of groups was tested using Fisher’s post hoc test (the pairwiseNominalIndependence() method of the {rcompanion} package was used for this purpose).

## 3. Results

### 3.1. The Effect of Ankyloglossia on Tongue Function during Swallowing

The percentage of patients with ankyloglossia (study group, taking into account the severity of ankyloglossia) presenting the persistent infantile type of swallowing and the mature type of swallowing and patients with a normal frenulum (control group) are presented in Table 2. Fisher’s post hoc test showed a significant relationship between pairs of groups: mature type of swallowing vs. infantile type of swallowing (Fisher *p* < 0.001, V = 0.39). Fisher’s exact test for the entire study population showed a significant relationship between swallowing type and severity of ankyloglossia (*p* < 0.05). Among subjects with the infantile type of swallowing, only 24.4% had a normal length of the tongue frenulum, and the majority were people with ankyloglossia. Among subjects presenting the mature type of swallowing, a significantly higher percentage had a normal frenulum.

### 3.2. The Effect of Ankyloglossia on Tongue Mobility

The results show that ankyloglossia reduced tongue mobility, but only in patients with moderate and severe ankyloglossia. All patients with reduced tongue mobility had ankyloglossia (over 70% were those with severe ankyloglossia, and the latter were patients with moderate ankyloglossia). The results showed that the shorter tongue frenulum, the higher the percentage of patients with limited tongue mobility. All subjects from the control group and all those diagnosed with mild ankyloglossia showed normal tongue mobility. The results are presented in Table 3. Fisher’s exact test for the entire study population showed a significant relationship between tongue mobility and the severity of ankyloglossia.

### 3.3. The Effect of Ankyloglossia on the Presence of Dysglossia

The results of the present study show that ankyloglossia had an impact on articulation disorders, such as “r” /r/ problems or “s” /s/, “sz” /ʂ/ problems. Problems with the Polish sound “r” accounted for a higher proportion of dysglossia than problems with “r” /r/ and “s” /s/, “sz” /ʂ/ together, and the least common isolated problem related to “s” /s/, “sz” /ʂ/ pronunciation disorders. With higher classes of ankyloglossia, more problems with the isolated “r” /r/ sound were found.

The present research showed that patients with a normal tongue frenulum showed a lower tendency to present with speech abnormalities such as lisping and rhotacism. Among patients with correct pronunciation, more than a half were subjects without ankyloglossia. Rhotacism most often concerned patients with the II and III classes of ankyloglossia. Of the 14 subjects showing simultaneous rhotacism and lisping, almost a half had a normal length of the tongue frenulum. The percentage of subjects with dysglossia increased with the increase in the severity of ankyloglossia. These differences were statistically significant. The results of the study are presented in Table 4. Fisher’s post hoc test showed a significant relationship between individual pairs of groups: normal speech vs. rhotacism (*p*_Fisher_ < 0.001, V = 0.49). Fisher’s exact test showed a statistically significant relationship between the correctness of speech and the class of ankyloglossia (*p* < 0.05).

### 3.4. The Effect of Shortened Tongue Frenulum on the Occurrence of Malocclusion or Crowding

The results obtained in the control group and in the study group, divided into individual severity of ankyloglossia, are presented in Table 5. Fisher’s exact test for the entire study population showed a significant relationship between the presence of malocclusion and the severity of ankyloglossia (*p* < 0.05). The present results clearly indicate a relationship between the degree of ankyloglossia and the presence of malocclusion.

In patients with normal occlusion, the majority were subjects with a normal tongue frenulum, and only 21.6% were diagnosed with ankyloglossia. The percentages of subjects with and without ankyloglossia among patients with malocclusion were distributed differently. Only 38% were subjects with a normal tongue frenulum, and the majority were patients with ankyloglossia.

### 3.5. The Effect of Ankyloglossia on Periodontal Status

In the present study, no abnormalities in the periodontium in the area of the lower central incisors were found in any of the examined subjects; therefore, no analysis of the relationship between this parameter and other examined parameters was performed.

## 4. Discussion

The present findings are consistent with previous studies referring to the influence of ankyloglossia on primary tongue functions, such as tongue mobility, eating, drinking, speech, as well as on malocclusion (including distal occlusion, mesial occlusion, crossbite, open bite and crowding) [20,27,28,29,30,31]. However, no relationship between short tongue frenulum and periodontal disorders in the region of the lower central incisors found in the present study is contrary to case reports published in the literature [32].

### 4.1. Effect of Shortened Tongue Frenulum on Tongue Function during Swallowing

The occurrence of a short tongue frenulum in subjects with persistent infantile swallowing in the present study supports the results by Huang et al. [33] on 27 children aged 2–16 with a short tongue frenulum and sleep-disordered breathing (including snoring, reduced blood saturation and sleep apnea). The study cited found that children with ankyloglossia had a disturbed swallowing pattern, but the type of disorder was not defined.

The background for the lack of transformation of infantile swallowing into mature swallowing seems to be reduced vertical tongue movement, which is necessary for mature swallowing. This hypothesis is consistent with the present study, as reduced tongue mobility was related to ankyloglossia and infantile swallowing.

Thus, in the present study, no subject with reduced tongue mobility was found in the control group or in the group with mild ankyloglossia, whereas in moderate ankyloglossia, among subjects with an infantile swallowing pattern, reduced tongue mobility was found in 23% of subjects.

Moreover, the present findings that reduced tongue mobility was stated in 31.1% of the subjects with the infantile swallowing type and only in 2.4% of the subjects with normal swallowing indicates an important role of normal tongue mobility in the maturation of swallowing.

In the paper on the diagnostics and surgical treatment of ankyloglossia in children, Kotlow [32] discusses the influence of ankyloglossia on swallowing.

The study by Ruffoli et al. on 200 children aged 6–12 years with ankyloglossia found infantile swallowing in 45% of subjects [20]. The authors of the paper cited reported no correlation between ankyloglossia and persistent infantile swallowing, although no control group was included in their study. Wright [34], in a study on 287 patients with ankyloglossia, found infantile swallowing only in 13%. In the present study, among 86 subjects with ankyloglossia 34 (39.5%) demonstrated an infantile type of swallowing. This result is similar to observations made by Ruffoli et al. [20].

### 4.2. Effect of Ankyloglossia on Tongue Mobility

In our comprehensive study, we established a profound relationship between ankyloglossia and impaired tongue mobility, which serves as the foundational factor behind a range of associated dysfunctions and abnormalities. Understanding the etiology of this limitation is paramount, given its implications, including challenges in transitioning from infantile to mature swallowing patterns, difficulties in articulation leading to dysglossias and functional malocclusion. Our investigation revealed that it is primarily the significant shortening of the frenulum of the tongue, categorized as moderate and severe ankyloglossia, that exerts a notable constraint on tongue mobility. This assertion supports the study by Kotlow, who meticulously examined a cohort of 322 children, aged from 18 months to 14 years, with varying degrees of tongue frenulum shortening [32]. He classified them into four distinct classes, based on the length of the free part of the tongue, measured from the distal attachment of the frenulum to the tip of the tongue. Class I corresponded to mild ankyloglossia (16 to 12 mm); Class II denoted moderate ankyloglossia (11 to 8 mm); Class III signified severe ankyloglossia (7 to 3 mm); and Class IV represented complete ankyloglossia (less than 3 mm). In his assessment, Kotlow considered nine critical criteria, encompassing abilities such as proper breastfeeding in infants, tongue protrusion in older children, correct swallowing (without tongue protrusion during the act) and unimpeded speech (though specific articulation issues were not explicitly detailed). This classification system aimed to delineate the indications for surgical intervention, specifically frenulectomy, to address tongue frenulum length. Kotlow’s findings illuminated the necessity for surgical intervention in all patients classified in Class IV and a substantial proportion of those classified as Class III ankyloglossia, as they exhibited substantial limitations in tongue mobility. Nevertheless, an intriguing revelation emerged from his research: Some children with severe ankyloglossia displayed remarkable motor compensations, enabling them to execute prescribed tongue movements despite their constrained mobility. These compensatory movements, however, were characteristically effortful. Consequently, it is reasonable to conclude, as our study affirms, that patients with only a slight shortening of the tongue frenulum do not experience significant restrictions in tongue mobility. Corroborating our findings, a prior study conducted by Ruffoli et al. found tongue mobility limitations in only 13% of 200 children with ankyloglossia [20]. Similarly, Lalakea et al. examined 14 patients with ankyloglossia and identified limited mobility in 57% of cases [35]. In 2020, Messner et al. conducted a comprehensive review of English-language literature, encompassing major publications concerning the diagnosis, symptoms and indications for surgical treatment of ankyloglossia in children aged 0 to 18. Their analysis in PubMed, EMBASE and Web of Science led them to conclude that ankyloglossia in older children and adolescents, owing to its undeniable impact on tongue mobility, may induce social challenges, including feelings of self-consciousness stemming from the inability to perform tasks like licking lips or cleaning teeth with the tongue [36]. In summary, our study sheds light on the intricate relationship between ankyloglossia and tongue mobility, offering valuable insights into the clinical implications and potential interventions for this condition.

### 4.3. Effect of Ankyloglossia on Speech Abnormalities and Dysglossia Prevalence

This study unveiled a statistically significant correlation between the presence/severity of ankyloglossia and the emergence of articulation abnormalities, notably including speech disruptions like rhotacism and lisping. These dyslalias appeared both in isolation, as single dysglossias, such as rhotacism lisping, and more occasionally in combination. The findings resonate with observations made by Polish speech therapists regarding the impact of ankyloglossia on the incidence of dysglossia, attributable to constraints in the vertical movements of the tongue associated with this anatomical anomaly. In the case of intricate tongue movements, as required for producing sounds like Polish “r,” the difficulties extend beyond merely lifting the tongue to make contact with the front part of the hard palate; they also encompass the ability of the tongue tip to vibrate. This could explain a higher percentage of patients experiencing rhotacism across all levels of ankyloglossia severity in the present study. In the studies by Ostapiuk [37,38,39,40] on speech quality in Polish individuals with ankyloglossia, 65% to 98% of individuals with a shortened tongue frenulum failed to correctly articulate Polish sounds such as “r /r/, sz /ʂ/, ż /ʂ/, cz /t͡ʂ/, dż /d͡ʒ/, ś /ɕ/, ź /ʑ/, ć /t͡ɕ/, dź /d͡ʑ/, ń /ɲ/, j /j/, s /s/, z /z/, c /t͡s/, dz /d͡z/”. The results also revealed that 80% of patients with frenulum shortening struggled with the correct pronunciation of the “l” /l/ sound. Interestingly, this percentage decreased with age, with 100% of children under 4 years and 58% of individuals over 14 years mispronouncing this sound. Ostapiuk further estimated that the percentage of individuals with ankyloglossia exhibiting phonetic inaccuracies in other sounds, including “t /t/, d /d/, n /n/, f /f/, w /v/, k /k/, g /g/, ch /h/, ł /w/,” was significantly lower, ranging from 6% to 11% [37,38,39,40]. The extent of this issue becomes even more apparent when considering the percentage of patients with ankyloglossia among individuals with otherwise normal speech but diagnosed with dyslalia, as identified in our study. Marchesan conducted an analysis of speech disorders among 127 patients in Brazil ranging from 5 to 62 years of age and found that 48.8% exhibited speech difficulties. These often included omissions or substitutions of the “r” /r/ sound, conflation of the “z” /z/ and “s” /s/ sounds and of the “r” /r/ and “z” sounds, as well as occasional lisping [41]. In Taiwan, Huang, in collaboration with researchers from the USA, examined 27 children with ankyloglossia and concurrent sleep-disordered breathing (SDB), determining that 48% of them encountered speech issues. However, the study did not specify the sounds associated with these problems [33]. In the previously mentioned study by Wright involving 287 patients with ankyloglossia, speech disorders were identified in 32% of cases. These issues encompassed articulation disorders and difficulties in the range of articulation that adversely affected speech intelligibility, although the specific sounds involved were not detailed [33]. Greek authors also noted in their review paper that individuals diagnosed with ankyloglossia encountered challenges with the articulation of sounds like “t, d, th, l, s” [42]. Some authors, such as Wang et al. [43], in a review article based on the analysis of 16 studies on the impact of ankyloglossia on articulation problems, concluded that there is no definitive correlation between tongue frenulum shortening and speech disorders. Notably, a study by Guilleminault et al. [44] measured the tongue frenulum using the Ruffoli method (the same method employed in this study) in 150 children aged 3 to 12 with sleep disorders. Among these, 63 were diagnosed with ankyloglossia, while 87 had a normal-length tongue frenulum. Within the ankyloglossia group, 49.2% of children (31 out of 63) exhibited speech issues, whereas none in the non-ankyloglossia group received such a diagnosis [44].

### 4.4. Effect of Ankyloglossia on Dental and Malocclusion Disorders

Our study unveiled a significant relationship between ankyloglossia and malocclusion or dental abnormalities, constituting 70.3% of the entire study population. This observation aligns with the epidemiology of malocclusion in Poland [45], where 62% of those with malocclusion or dental abnormalities exhibited a shortened tongue frenulum, while among individuals without these issues, ankyloglossia was found in only 22%. A comparative analysis between groups with and without ankyloglossia yielded statistically significant results regarding the relationship between the presence of a shortened tongue frenulum and the prevalence of malocclusion. In the ankyloglossia group, individuals with malocclusion accounted for 87.2%. Ruffoli et al. also reported occlusal abnormalities in 61.5% of 200 children with ankyloglossia [20].

In contrast, Yoon et al. conducted a study involving 302 patients aged 6 to 67 with diagnosed ankyloglossia, categorized into four classes according to Kotlow. Orthodontic assessments, plaster models and cephalograms were analyzed. Their study did not reveal statistically significant associations between skeletal malocclusions based on Angle’s classification and ankyloglossia. Furthermore, no statistically significant changes in jaw structure related to the degree of tongue frenulum shortening, such as reduced jaw width-to-length ratio or decreased intermolar and intercanine distances, were identified [46]. However, Pompeia et al. emphasized, in their review paper, that ankyloglossia negatively impacts facial skeletal growth and development [47].

Srinivasan et al. examined 57 Indian patients diagnosed with ankyloglossia and skeletal malocclusions, comparing them with a group of 60 individuals without ankyloglossia but with skeletal malocclusions. Ankyloglossia in patients was classified as per Kotlow’s four classes. The study found that moderate ankyloglossia (equivalent to mild ankyloglossia in our study) was associated with the most common defects, particularly a transverse maxillary deficiency at the canine and molar levels, consistent with Yoon’s findings. Conversely, in more severe forms of ankyloglossia, specifically severe and total ankyloglossia according to Kotlow (corresponding to mild ankyloglossia in our study), statistically significant changes in mandibular angles and an increased occurrence of deep bite were observed [48]. Furthermore, Jang et al. demonstrated the influence of ankyloglossia on skeletal dysplasias within the maxilla and mandible. They examined 150 individuals divided into three groups based on skeletal classes according to Angle. The study measured tongue frenulum length and found that in the group with skeletal malocclusions (indicating a neutral relationship between maxilla and mandible bone bases), the average frenulum length was 3.3 mm. In the group with skeletal Class II (indicating the mandible positioned posteriorly to the maxilla), the average frenulum length was 3.3 mm. In contrast, the group with skeletal Class III (indicating an anterior relation of the mandible to the maxilla) exhibited a longer average frenulum length, 4.9 mm. The study revealed that tongue frenulum length in ankyloglossia was proportionate to mandibular body length, potentially explaining the longest average frenulum length in the group with anterior malocclusions [27].

Meenakshi et al. studied 30 individuals aged 12 to 16 diagnosed with ankyloglossia [30]. They categorized them into three groups, each with a different skeletal class according to Angle: Class I, Class II and Class III. Tongue frenulum length was measured using plaster models (cast from alginate impressions) of the lower tongue surface. The third group exhibited the longest average tongue frenulum length (5.27 mm), while the first and second groups presented lengths of 3.96 mm and 4.08 mm, respectively. The results suggest that milder ankyloglossia may predispose individuals to mandibular prognathism through increased tongue pressure on the lower dental arch in both transverse and sagittal dimensions. Conversely, more severe forms of ankyloglossia may contribute to alveolar retrognathism [30]. As previously mentioned, Guilleminault et al. examined 150 children and found that in the ankyloglossia group, 56 out of 63 children were diagnosed with palatal stenosis, while in the non-ankyloglossia group, only 7 individuals exhibited palatal stenosis [44].

It seems that ankyloglossia may be an important factor contributing to altered growth and development leading to malocclusions, which require orthodontic treatment [49]. Thus, surgical correction of ankyloglossia may be indicated. However, it should be kept in mind that surgical treatment of ankyloglossia involves the section and disinsertion of the frenulum fibers through the injection of local anesthetic to allow for greater lingual mobility; possible complications are hypoesthesia, bleeding, complications linked to the migration of the anesthetic solution [50,51].

### 4.5. Effect of Ankyloglossia on Periodontium

This study found no adverse impact of ankyloglossia on the periodontium in the individuals under examination. Additionally, Suter et al. reported no discernible alterations in the periodontium within the lingual surfaces of the lower central incisors [7]. Conversely, in a study involving 322 children, Kotlow noted the presence of some alterations, although specific figures were not provided. These abnormalities primarily manifested as diastema, occurring in cases of severe ankyloglossia, where increased tension of the oral mucosa resulted from tongue movements. This increased tension led to tissue retraction behind the mandibular incisors and the development of diastema. Notably, the author did not document any other symptoms, such as recessions or dehiscences, in association with ankyloglossia [32].

## 5. Conclusions

1. A short tongue frenulum negatively influences swallowing and is associated with an “infantile swallowing pattern”.
2. Moderate or severe ankyloglossia significantly limits tongue mobility.
3. A short tongue frenulum negatively influences speech.
4. Ankyloglossia is associated with higher prevalence of malocclusion.

## Figures and Tables

**Table 1:** Distribution of the study and control groups according to sex.

Sex		Study Group		Control Group	Total
	Ankyloglossia Severity				
	Mild	Moderate	Severe		
Female	5 (5.2%)	21 (21.9%)	16 (16.7%)	54 (56.2%)	96
Male	5 (6.6%)	28 (36.8%)	11 (14.5%)	32 (42.1%)	76
Total	10 (5.8%)	49 (28.5%)	27 (15.7%)	86 (50%)	172

**Table 2:** Types of swallowing in the study and control groups with the results of the independence test.

Type of Swallowing		Study Group		Control Group	Total
	Ankyloglossia Severity				
	Mild	Moderate	Severe		
Infantile	5 (11.1%)	13 (28.9%)	16 (35.6%)	11 (24.4%)	45
Mature	5 (4%)	36 (28.6%)	11 (8.7%)	74 (58.7%)	126
Mixtus	0	0	0	1 (100%)	1
Total	10 (5.8%)	49 (28.5%)	27 (15.7%)	86 (50%)	172
Independence test results:		χ^2^ = 16.912, df = 6, V = 0.176, *p*_Fisher_ < 0.001			

**Table 3:** Tongue mobility in the study and control groups with the results of the independence test.

Tongue Mobility		Study Group		Control Group	Total
	Ankyloglossia Severity				
	Mild	Moderate	Severe		
Limited	0	5 (29.4%)	12 (70.6%)	0	17
Normal	10 (6.5%)	44 (28.4%)	15 (9.7%)	86 (55.5%)	155
Total	10 (5.8%)	49 (28.5%)	27 (15.7%)	86 (50%)	172
Independence test results:		χ^2^ = 46.743, df = 3, V = 0.521, *p*_Fisher_ < 0.001			

**Table 4:** Speech in the study and control groups with the results of the independence test.

Speech		Study Group		Control Group	Total
	Ankyloglossia Severity				
	Mild	Moderate	Severe		
Normal	8 (7.3%)	27 (24.5%)	6 (5.5%)	69 (62.7%)	110
Rhotacism	1 (2.1%)	18 (38.3%)	18 (38.3%)	10 (21.3%)	47
Rhotacism + lisping	1 (7.1%)	4 (28.6%)	3 (21.4%)	6 (42.9%)	14
Lisping	0	0	0	1 (100%)	1
Total	10 (5.8%)	49 (28.5%)	27 (15.7%)	86 (50%)	172
Independence test results:		χ^2^ = 39.131, df = 6, V = 0.275, *p*_Fisher_ < 0.001			

**Table 5:** Occurrence of malocclusion or crowding in the study and control groups with the results of independence test.

Crowding/Malocclusion		Study Group		Control Group	Total
	Ankyloglossia Severity				
	Mild	Moderate	Severe		
Absent	1 (2%)	8 (15.7%)	2 (3.9%)	40 (78.4%)	51
Present	9 (7.4%)	41 (33.9%)	25 (20.7%)	46 (38%)	121
Total	10 (5.8%)	49 (28.5%)	27 (15.7%)	86 (50%)	172
Independence test results:		χ^2^ = 24.147, df = 3, V = 0.375, *p*_Fisher_ < 0.001			

## Data Availability

The data presented in this study are available on request from the corresponding author. The data are not publicly available due to ethical restiction.
